# Supplementary material for: A Latent Inflammatory Tissue-State Variable Mechanistically Links Radiotherapy-Induced Immune Remodeling to Recurrent Tumor Permissiveness
Source: bioRxiv. 2026 Sep 17:2026.09.14.751444. Preprint. [Version 1] doi: 10.64898/2026.09.14.751444 (PMC13596334; doi:10.64898/2026.09.14.751444)
Supplement: Supplementary file 1 [file NIHPP2026.09.14.751444v1-supplement-1.pdf]

bioRxiv preprint doi: https://doi.org/10.64898/2026.09.14.751444; this version posted September 17, 2026. The copyright holder for this preprint (which was not certified by peer review) is the author/funder, who has granted bioRxiv a license to display the preprint in perpetuity. It is made available under a CC-BY 4.0 International license.

Supplementary Table S1. Parameters and Parameter Sources

**Parameter Name**					
**Macrophage Count**	**Time Post-Radiation (Days)**	**Nonrecurrent (Count per Field of View)**	$\frac{\text{Nonrecurrent}(t)}{\text{Nonrecurrent}(0)}$	**Recurrent (Count per Field of View)**	$\frac{\text{Recurrent}(t)}{\text{Nonrecurrent}(0)}$
**Parameter Description**	**0**	5.00	1.00	3.50	0.700
**F4/80+ macrophages found in the immunodeficient irradiated mammary fat pad of mice via immunohistochemistry**	**1**	6.41	1.28	4.43	0.866
**Source**	**5**	7.59	1.52	7.08	1.416
**Rafat *et al* 2018, Cancer Research**<br>**PMID: 29880480**	**10**	17.66	3.53	36.19	7.238

**Parameter Name**			
**Percent Macrophage Polarization**	**Family**	**Nonrecurrent (%)**	**Recurrent (%)**
**Parameter Description**	**F4/80+ Macrophages>M0**	90	85
**Polarization of macrophages at the irradiated mammary fat pad 10 days post-radiation via flow cytometry**	**F4/80+ Macrophages>M1**	5	10
**Source**	**F4/80+ Macrophages>M2**	5	5
**Hacker *et al* 2023, Cell Mol Bioengineering**<br>**PMID: 37810999**			
